# An Atypical Abdominal Presentation of Pancreatic Panniculitis in a Patient With Suspected Pancreatic Cancer: A Case Report

**DOI:** 10.7759/cureus.116124

**Published:** 2026-09-12

**Authors:** Takayuki Ota, Yosuke Suyama

**Affiliations:** 1 Gastroenterology, Rakuwakai Marutamachi Hospital, Kyoto, JPN

**Keywords:** abdominal panniculitis, fat necrosis, ghost adipocytes, pancreatic cancer, pancreatic enzymes, pancreatic panniculitis

## Abstract

Pancreatic panniculitis (PP) is an uncommon manifestation of pancreatic disorders and usually presents as painful erythematous subcutaneous nodules involving the lower extremities. We describe a 79-year-old man with a pancreatic head tumor clinically suspected to represent pancreatic cancer who developed a painful, erythematous, approximately 10-cm abdominal wall lesion. Contrast-enhanced computed tomography showed inflammatory changes involving the subcutaneous and abdominal wall adipose tissue. Despite the extensive lesion, pancreatic enzyme elevation was modest, with a lipase level of 157 IU/L and pancreatic amylase level of 86 IU/L. Malignant involvement and soft-tissue infection were initially considered because of the lesion's location and appearance. Histopathological examination demonstrated lobular panniculitis, fat necrosis, and anucleated adipocytes with thickened borders and eosinophilic material, consistent with ghost-like adipocytes of PP. Klebsiella pneumoniae was isolated from the aspirated material, raising the possibility of secondary bacterial infection. Cefmetazole was administered at 1 g twice daily for five days; subsequent susceptibility testing showed cefmetazole resistance. After the histopathological diagnosis of PP was established, symptomatic treatment with oral loxoprofen was initiated. The pain, erythema, and swelling gradually improved, and the lesion had markedly resolved by day 60, with corresponding improvement of the abdominal wall inflammatory changes on follow-up computed tomography. This case illustrates that PP may occur at an atypical abdominal site despite only modest pancreatic enzyme elevation and may mimic infection or malignant involvement. Histopathological evaluation of a subcutaneous lesion can be particularly valuable when clinical and biochemical findings are atypical, and a definitive pathological diagnosis of the underlying pancreatic lesion is not obtained.

## Introduction

Pancreatic panniculitis (PP) is a rare extra-pancreatic manifestation of pancreatic disease, reported in approximately 0.3%-3% of patients with pancreatic disorders [1-3]. It has been described in association with acute and chronic pancreatitis, pancreatic pseudocysts, and pancreatic neoplasms. Among malignant conditions, pancreatic acinar cell carcinoma has a particularly strong association with PP, although other pancreatic tumors have also been reported [1,4-6].

The usual clinical pattern consists of tender erythematous or violaceous subcutaneous nodules, predominantly on the lower extremities. Histologically, PP is characterized by predominantly lobular fat necrosis with an inflammatory infiltrate. A highly characteristic clue is the presence of anucleated necrotic adipocytes with thickened, shadow-like outlines, commonly termed ghost cells or ghost adipocytes [2,3,7].

Systemic release of pancreatic enzymes, particularly lipase, is considered central to pathogenesis, and substantial elevations in serum pancreatic enzymes are common in reported cases [1,4,5]. PP associated with malignancy has a particularly well-recognized relationship with acinar cell carcinoma (ACC), in which marked hyperlipasemia may occur; however, PP has also been reported with other pancreatic neoplasms [1,4-6]. Accordingly, pathological characterization of the underlying pancreatic tumor, for example by endoscopic ultrasound-guided fine-needle aspiration (EUS-FNA), can be important when clinically feasible. Nevertheless, the association between serum enzyme concentration and the development of PP is not absolute. In addition, lesions outside the lower extremities, including truncal and abdominal lesions, have been described [1,8,9]. At such atypical sites, PP may clinically resemble cellulitis, direct malignant extension, or cutaneous metastatic disease, making tissue diagnosis particularly important.

We report histologically confirmed PP in a patient with a pancreatic head tumor clinically suspected to represent pancreatic cancer but without pathological confirmation of the primary tumor. The case was unusual because the panniculitis manifested as a large abdominal lesion, pancreatic enzyme elevation was modest, and aspiration culture yielded Klebsiella pneumoniae, complicating the distinction between PP and bacterial soft-tissue infection. The abdominal localization also raises questions regarding whether local anatomical factors, in addition to systemic enzyme dissemination, may have contributed to the distribution of the lesion.

## Case presentation

A 79-year-old man initially presented to our hospital in late March with sudden epigastric pain. His medical history included hypertension, premature ventricular contractions, and previous gastric bypass surgery. His regular medication was amlodipine besylate 10 mg/day.

Contrast-enhanced computed tomography demonstrated an approximately 30-mm hypovascular mass in the pancreatic head (Figure 1) with obstructive jaundice. Direct tumor invasion of the duodenum caused severe luminal narrowing, making transpapillary biliary drainage difficult. Percutaneous transhepatic biliary drainage was therefore performed.

**Figure 1:**
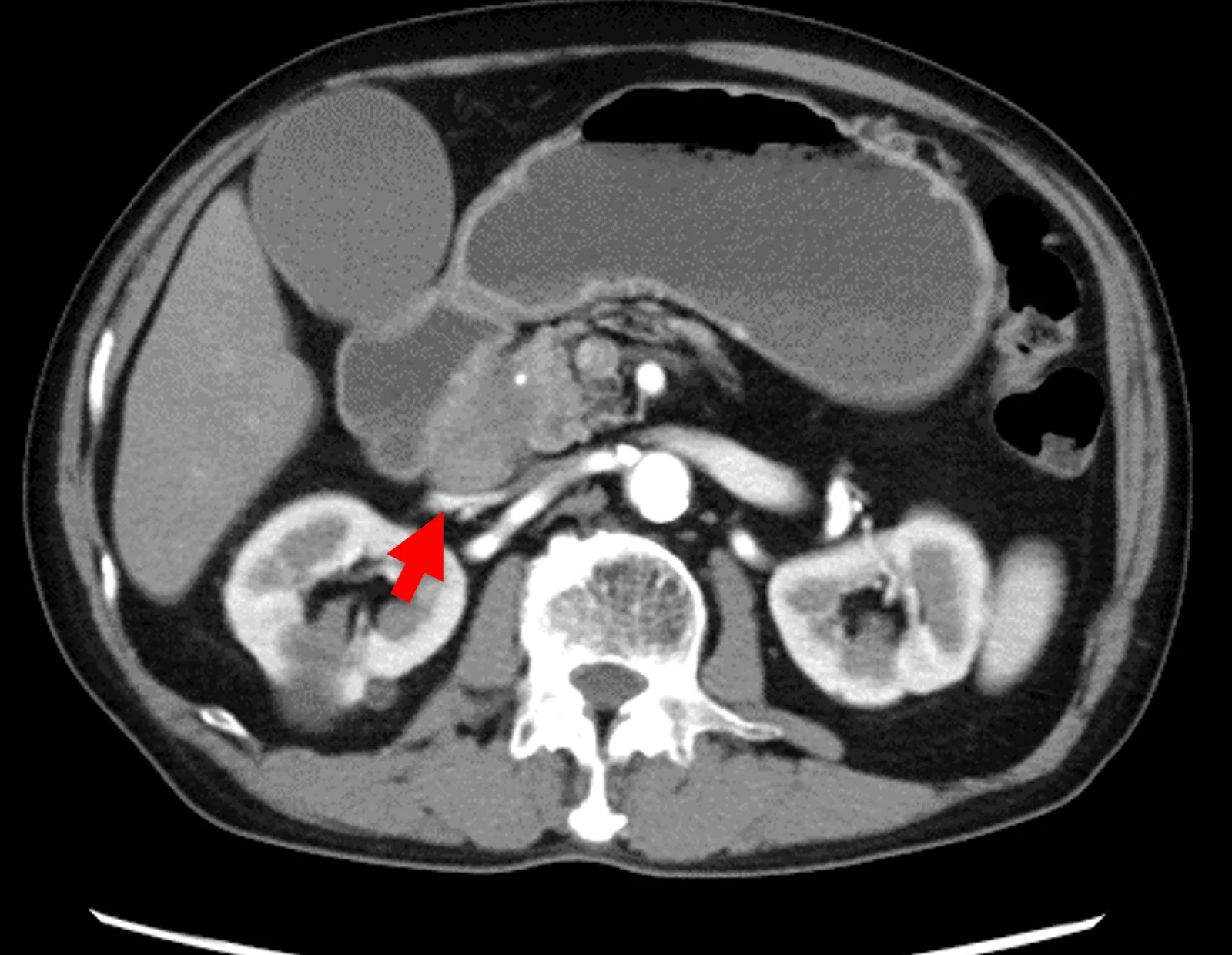
Pancreatic head tumor on contrast-enhanced computed tomography Contrast-enhanced computed tomography demonstrates an approximately 30-mm hypovascular mass in the pancreatic head (arrow).

The clinical and radiological findings were highly suggestive of pancreatic cancer. Tissue sampling of the pancreatic mass, including EUS-FNA, was considered; however, the patient declined invasive diagnostic procedures and antineoplastic treatment and chose best supportive care. Histopathological confirmation of the pancreatic tumor was therefore not obtained, and its histological subtype remained undetermined. He was subsequently followed as an outpatient with the percutaneous drainage catheter in place.

Approximately three months after the initial presentation, in mid-June, the patient developed a painful mass in the upper abdomen and was admitted for evaluation. His temperature was 36.9°C, pulse rate was 70 beats/min, blood pressure was 130/78 mmHg, and oxygen saturation was 97% on room air. Physical examination revealed a well-demarcated, erythematous, swollen, elevated lesion approximately 10 cm in diameter in the midline epigastric region (Figure 2A). The lesion was tender. No peripheral edema was present.

**Figure 2:**
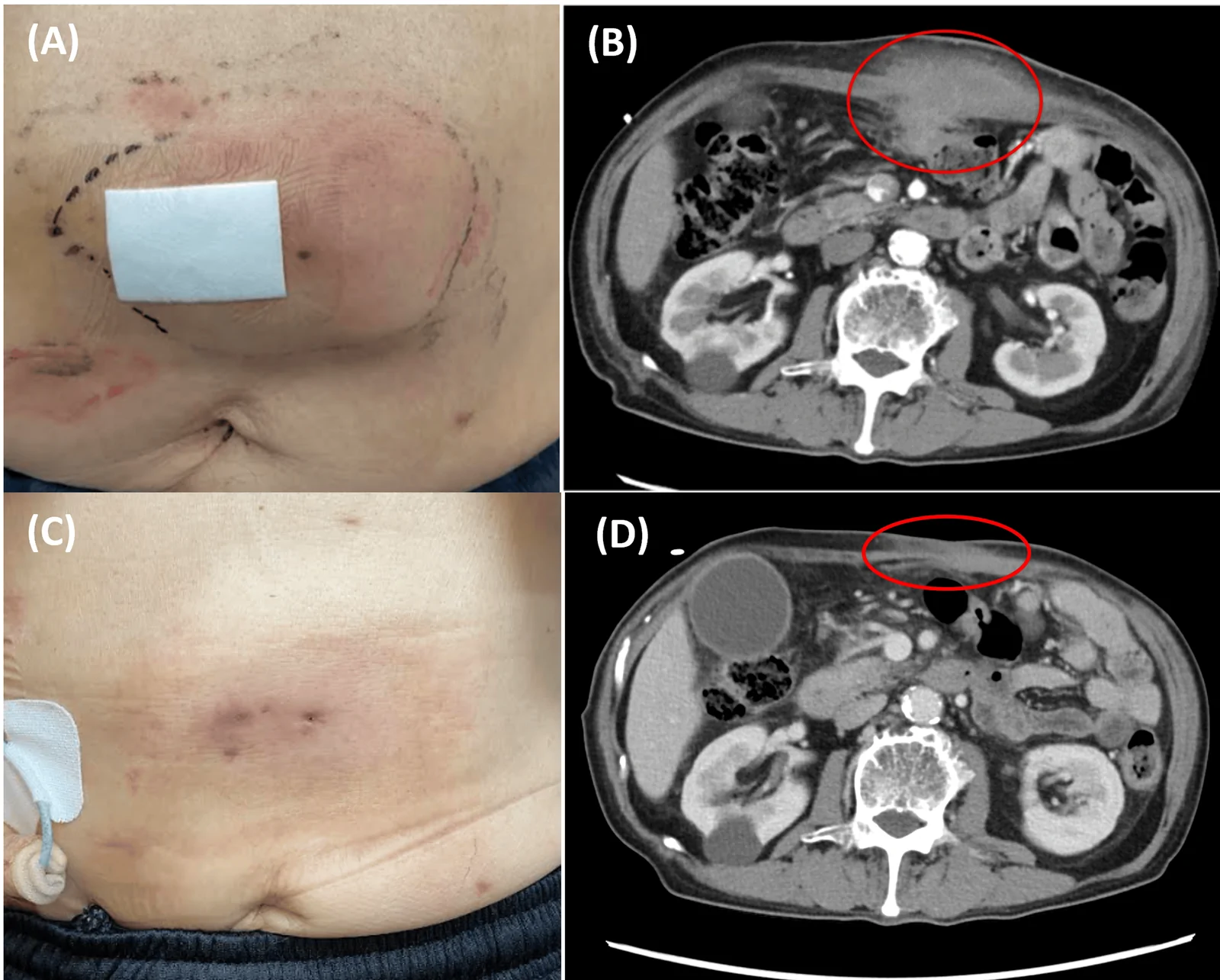
Clinical and radiological course of the abdominal wall lesion (A) Clinical photograph at the onset of the abdominal wall lesion showing an approximately 10-cm erythematous and swollen lesion in the upper abdomen. (B) Contrast-enhanced computed tomography (CT) at the onset of the lesion showing inflammatory changes extending from the subcutaneous tissue through the abdominal wall into the intra-abdominal adipose tissue (red circle). (C) Clinical photograph on day 60 showing marked resolution of the erythema and swelling, with only localized residual discoloration. (D) Follow-up contrast-enhanced CT on day 60 showing marked improvement of the inflammatory changes in the abdominal wall and surrounding adipose tissue (red circle).

Laboratory findings at admission are summarized in Table 1. 

**Table 1:** Laboratory findings at admission AST, aspartate aminotransferase; ALT, alanine aminotransferase; BUN, blood urea nitrogen; CRP, C-reactive protein; DUPAN-2, Duke pancreatic monoclonal antigen type 2; SPan-1, s-pancreas-1 antigen; CA19-9, carbohydrate antigen 19-9.

Parameter	Value	Unit	Reference range
White blood cell count	7800	/μL	4,000–8,000
Hemoglobin	10.7	g/dL	11.5–15.5
Platelet count	17.6	×10⁴/μL	150,000–350,000
Neutrophils	5700	/μL	46–60
Lymphocytes	1560	/μL	32–48
Total protein	7.1	g/dL	6.5–8.2
Albumin	3.6	g/dL	4.1–5.1
Total bilirubin	0.6	mg/dL	0.4–1.2
AST	22	IU/L	5–35
ALT	14	IU/L	1–40
Lipase	157	IU/L	13–60
Pancreatic amylase	86	IU/L	26–80
BUN	15.4	mg/dL	8–20
Creatinine	0.61	mg/dL	0.4–1.1
CRP	8.61	mg/dL	0–0.24
Sodium	137	mEq/L	135–147
Chloride	99	mEq/L	96–108
Potassium	3.2	mEq/L	3.5–4.6
Calcium	9.4	mg/dL	8.8–11.0
DUPAN-2	2000	U/mL	0–150
SPan-1	380	U/mL	0–30
CA19-9	2380	U/mL	0–37

Contrast-enhanced computed tomography obtained after development of the abdominal lesion showed increased attenuation of adipose tissue extending from the subcutaneous tissue through the abdominal wall toward the intra-abdominal fat (Figure 2B). In the setting of suspected pancreatic malignancy, malignant involvement of the abdominal wall was considered. The erythema, swelling, tenderness, and inflammatory imaging changes also raised concern for bacterial cellulitis or another soft-tissue infection.

Aspiration of the lesion was performed, and culture of the aspirated material yielded Klebsiella pneumoniae. Cefmetazole was administered at 1 g twice daily (2 g/day) for five days, together with topical treatment. Subsequent antimicrobial susceptibility testing showed that the isolate was resistant to cefmetazole. A later aspiration culture was negative.

Histopathological examination of tissue obtained by ultrasound-guided needle biopsy of the abdominal lesion showed prominent inflammatory cell infiltration centered on the fat lobules, disruption of normal lobular architecture, and scattered foci of fat necrosis. Some adipocytes had lost their nuclei and showed thickened cell borders with a central eosinophilic material, producing a ghost-like appearance (Figure 3). These findings supported the diagnosis of PP.

**Figure 3:**
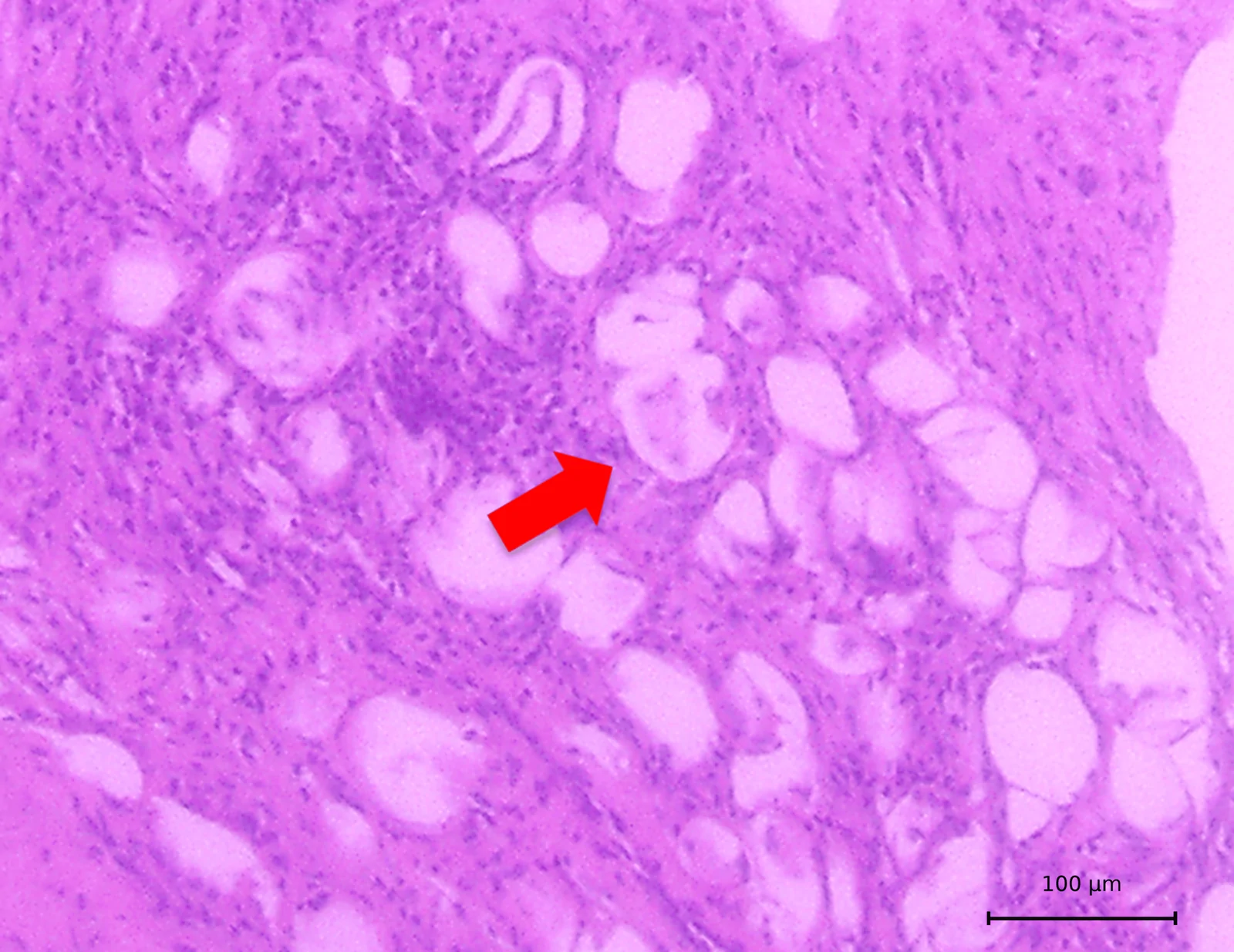
Histopathological findings of pancreatic panniculitis Hematoxylin and eosin staining of tissue obtained by ultrasound-guided needle biopsy of the abdominal wall lesion demonstrates lobular panniculitis with fat necrosis and characteristic ghost-like adipocytes. The affected adipocytes show loss of nuclei, thickened cell borders, and eosinophilic material (red arrow), findings consistent with pancreatic panniculitis. Original magnification, ×200; scale bar = 100 μm.

The positive culture suggested that a localized bacterial infection may have coexisted with PP. However, blood cultures were negative, the patient remained afebrile, and he did not develop leukocytosis. In addition, the isolate was resistant to cefmetazole, making bacterial infection alone an insufficient explanation for the subsequent clinical course.

After the histopathological diagnosis of PP was established, oral loxoprofen was initiated at three tablets per day in three divided doses as anti-inflammatory and analgesic treatment. Following initiation of loxoprofen, the pain, erythema, and swelling gradually subsided. By day 60, the abdominal lesion had markedly resolved, with only localized residual discoloration, and follow-up contrast-enhanced computed tomography demonstrated marked improvement of the inflammatory changes in the abdominal wall and surrounding adipose tissue (Figures 2C, 2D). At that time, the white blood cell count had decreased to 4,400/μL and CRP to 0.03 mg/dL. In contrast, tumor markers remained markedly elevated: CA19-9 was 2,310 U/mL, DUPAN-2 was 2,300 U/mL, and SPan-1 was 540 U/mL. Lipase and pancreatic amylase were not remeasured after the onset of the abdominal lesion.

## Discussion

The present case differs from the usual clinical picture of PP in three clinically relevant respects: the lesion was centered on the abdominal wall rather than the lower extremities, circulating pancreatic enzyme levels were only modestly elevated, and a positive bacterial culture complicated interpretation of the lesion.

PP is uncommon even among patients with pancreatic disease. Recent reviews and a multicenter clinicopathological series emphasize that the lower extremities are the dominant site of involvement and that histopathology often provides the decisive diagnostic clue [1,2]. Abdominal and truncal lesions are recognized but are less typical [1,8]. In our patient, the abdominal location and proximity to the pancreatic lesion raised concern for malignant extension, while the erythematous and tender appearance together with inflammatory changes on computed tomography suggested a soft-tissue infection. The mechanism responsible for the unusual midline upper abdominal localization remains uncertain. Systemic dissemination of pancreatic enzymes remains the established mechanism of PP, but local anatomical factors may also have influenced the distribution in this patient. His previous gastric bypass surgery, the presence of a percutaneous transhepatic biliary drainage catheter, and the proximity of the lesion to the peripancreatic region raise the possibility of altered local tissue or lymphatic conditions. Direct extension or local lymphatic spread from the peripancreatic region cannot be excluded; however, these mechanisms remain speculative, and the histopathological findings favored PP rather than direct tumor involvement.

The modest pancreatic enzyme elevation was another atypical feature. Published tumor-associated cases, particularly those associated with ACC, frequently show striking hyperlipasemia, sometimes reaching several thousand units per liter [4-6]. Because the pancreatic tumor in our patient was not histologically characterized, its subtype cannot be determined. The clinical and radiological appearance was considered suspicious for conventional pancreatic cancer, but ACC with limited enzyme production cannot be completely excluded. Likewise, localized pancreatic inflammation or pancreatitis associated with the tumor could theoretically have contributed to pancreatic enzyme release. Nevertheless, serum enzyme levels should not be used in isolation to exclude PP. A single measurement may not reflect earlier tissue exposure to circulating enzymes, and host or local tissue factors may influence susceptibility to enzymatic fat injury. In this case, the combination of lobular panniculitis, fat necrosis, and ghost-like adipocytes was more diagnostically informative than the magnitude of enzyme elevation.

The prevailing pathophysiological model proposes that pancreatic enzymes enter the systemic circulation and reach distant adipose tissue, where lipase-mediated triglyceride hydrolysis causes fat necrosis and saponification; proteolytic enzymes may facilitate tissue penetration by increasing vascular permeability [3,7]. The fact that PP develops in only a minority of patients with pancreatic disease suggests that additional factors probably contribute.

Histopathological examination was central to diagnosis. PP classically shows a predominantly lobular panniculitis with enzymatic fat necrosis. Necrotic adipocytes may lose their nuclei while retaining thickened outlines and granular or eosinophilic material, producing the characteristic ghost-cell appearance [2,3,7]. These findings were present in our patient and argued strongly against uncomplicated cellulitis or cutaneous tumor involvement.

The isolation of K. pneumoniae from the aspirated lesion adds an unusual diagnostic layer. A true secondary localized bacterial infection of necrotic tissue could not be excluded, although contamination also remains possible. Blood cultures were negative, and the absence of fever or leukocytosis, the characteristic histopathology, and continued regression despite cefmetazole resistance make infection alone unlikely to account for the lesion. A later aspiration culture was negative. The most cautious interpretation is that PP was the primary pathological process with possible superimposed bacterial infection.

Treatment of PP is directed principally at the underlying pancreatic disorder [1,3]. Successful control of pancreatitis, pancreatic duct disruption, pseudocysts, or treatable pancreatic tumors is often followed by improvement in skin lesions. Supportive anti-inflammatory therapy has also been used. In the recent scoping review, nonsteroidal anti-inflammatory drugs (NSAIDs) were administered in a small minority of reported cases [1], and previous case reports have described resolution of dermatological manifestations in patients treated with NSAIDs as part of a combined therapeutic regimen [10]. However, evidence that NSAIDs themselves alter the natural course of PP is limited, and a specific therapeutic effect of loxoprofen has not been established. In our patient, loxoprofen was started only after histopathological confirmation of PP, after which the pain, erythema, and swelling gradually improved. By day 60, the cutaneous lesion and abdominal wall inflammatory changes on computed tomography had markedly improved, and CRP had decreased from 8.61 to 0.03 mg/dL. In contrast, the tumor markers remained markedly elevated, suggesting that resolution of the panniculitis did not simply parallel improvement of the underlying pancreatic tumor. Nevertheless, causality cannot be established. In addition to the anti-inflammatory and analgesic effects of loxoprofen, transient subsidence of pancreatic inflammation or enzyme release, spontaneous resolution of the panniculitis, and the preceding short course of cefmetazole and topical therapy may have contributed. Because lipase and pancreatic amylase were not serially measured, the relationship between pancreatic enzyme activity and resolution of the lesion could not be assessed.

This case has limitations. The pancreatic tumor was not histologically confirmed because, after tissue sampling such as EUS-FNA was considered, the patient declined invasive diagnostic procedures and antineoplastic treatment in accordance with his goals of care. Therefore, the histological subtype of the pancreatic neoplasm cannot be determined, and ACC with limited enzyme production cannot be definitively excluded. In addition, serial measurements of lipase and pancreatic amylase were unavailable after onset of the abdominal lesion, limiting assessment of the relationship between pancreatic enzyme activity and clinical resolution. The clinical significance of the positive K. pneumoniae culture also cannot be established with certainty. We therefore describe it as a possible secondary infection rather than attributing the lesion primarily to infection.

PP should remain in the differential diagnosis when a patient with pancreatic disease develops an unexplained painful inflammatory lesion of subcutaneous tissue, even when the lesion is abdominal and pancreatic enzyme levels are not dramatically elevated. When the clinical findings overlap with infection or malignant involvement, biopsy of the subcutaneous lesion and careful histopathological assessment may be decisive, particularly when definitive pathological diagnosis of the primary pancreatic lesion is difficult or cannot be obtained.

## Conclusions

PP usually presents as painful subcutaneous nodules of the lower extremities and is often accompanied by substantial pancreatic enzyme elevation. This case demonstrates that PP can instead present as a large abdominal wall lesion with only modest enzyme elevation, creating diagnostic overlap with soft-tissue infection and malignant involvement.

Recognition of lobular fat necrosis and ghost-like adipocytes on histopathological examination established the diagnosis despite atypical clinical and biochemical findings. In patients with pancreatic disease, PP should therefore remain in the differential diagnosis of otherwise unexplained painful inflammatory lesions of subcutaneous tissue. Biopsy of a subcutaneous lesion can be particularly useful for diagnosis and differential diagnosis when definitive pathological characterization of the primary pancreatic lesion is difficult or cannot be obtained.
